# Operative Procedure for Occluded Gall Bladder

**Published:** 1885-03-20

**Authors:** J. McF. Gaston

**Affiliations:** Professor of the Principles and Practice of Surgery


### Southern Medical Record.

EDITORS:

THOMAS S. POWELL, M. D. R. C. WORD, M. D.

*R. C. WORD, M.D., Managing Editor.*

☞ All Communications and Letters on Business connected with the **Record** must be addressed to the Managing Editor.

Vol. XV. ATLANTA, GA., MARCH 20, 1885. No. 3.

### ORIGINAL AND SELECTED ARTICLES.

### OPERATIVE PROCEDURE FOR OCCLUDED GALL-BLADDER.

An Autopsic Lecture in the amphitheatre of the Southern Medical College, Atlanta, Georgia, February 5, 1885,

By J. McF. Gaston, M. D.,

Professor of the Principles and Practice of Surgery.

Gentlemen: The body which lies before you, with the pathological specimens presented, will serve to demonstrate my mode of uniting the gall-bladder and the duodenum for the relief of obstruction in the gall duct. This patient died with obscure symptoms of occlusion in the outlet for the bile, and, as you see, the exposed gall-bladder is somewhat elongated and dilated beyond its normal proportions. We cannot perceive simply by external manipulation whether the ductus choledochus is entirely impermeable, but you observe that the duodenum is bound by adhesions to the transverse colon, and it is requisite to tear up these attachments, resulting from previous inflammation, to admit of a proper view of the relations of the duodenum to the pancreas and to the common duct. I now select a portion of the duodenal wall opposite, but somewhat below, the entrance of the bile duct, and pinch up with my thumb and forefinger a small doubling of the tissues which is transfixed with the curved needle, armed with an ordinary silk ligature. As is seen, this surface of the duodenum lies in close proximity to the lower and anterior surface of the gall-bladder ; so that pinching up its wall in the same manner, the needle with the ligature is also carried through its tissues ; and, drawing upon the two ends of the ligature, it is evident that it passes into the cavity of each to a very slight extent. This ligature being now freed from the needle, the surfaces of the duodenum and the gall-bladder, included within the loop are brought into immediate contact ; and they are secured together by knotting the ligature in such manner that the knot made shall press upon the duodenal wall. This apparently trivial step is indicated to ensure the passage of the loop into the cavity of the duodenum when it has cut its way through the conjoined tissues. In the first experiments, made for effecting this junction, a circular row of stitches with catgut was made to connect the serous membranes of the gall bladder and the duodenum, under the impression that this would be requisite to effect necessary adhesive inflammation between them. But it was evident that undue inflammtory action resulted from this, and by observation of the effects, in my last experiment, it was found that this adhesive inflammation followed the insertion of a single ligature ; so that the operation for securing the surfaces outside of this ligature is not desirable in any point of view. So far, then, as this proceeding is expected to effect an opening from the gall-bladder into the duodenum, and to insure the union of their outer walls around this orifice, nothing further is requisite than the passage of the ligature and knotting it as you see done in this instance. The ends are now cut off close to the knot, and you observe that it lies imbedded, as it were, in the wall of the duodenum, to insure its passing into this canal.

I have these specimens to show you to-day, illustrating the facts of fistulous communication between the gall-bladder and duodenum, by the cutting of the ligature, and the firm surrounding adhesions between their surfaces, from the inflammatory action set up in the serous membranes. One of these specimens was taken from a dog, which died on the fourteenth day, and with the handle of my scalpel passing through the duodenum, while the exterior wall of the gall-bladder is laid open so as to expose the cavity, you see the small slit in the septum formed by the agglutination of the two walls, which resulted from the action of the elastic ligature. In this case, the ligature cut its way through the tissues and lay within the gall-bladder.

The other specimen (which has also been preserved in alcohol) shows a similar opening between the gall-bladder and duodenum, in a dog which died on the eleventh day, the ligature having passed away in the intestinal canal.

I have here, also, a recent specimen of the entire liver, gall-bladder, and duodenum, which was removed yesterday afternoon from a dog that died under the administration of sulphuric ether by inhalation, while I was making an exploration of the results of my experiments made on the 20th and 28th of August in the past year. For five months this animal had been well and sprightly, after being subjected, in the first instance, to laparotomy and ligature of the walls of the gall-bladder and duodenum with a single silk ligature, and, eight days subsequently, a second laparotomy for the verification of the union of the two surfaces and the opening between the two cavities. The examination of this animal on yesterday was to ascertain the condition of these parts, after the lapse of such a length of time, with the intention of replacing the ligature if the orifice had closed up, and also expecting to ligate the common duct so as to thus prevent the escape of the bile by the natural channel. But, unfortunately, the animal succumbed from an over-dose of the anæsthetic soon after the peritoneal cavity was entered, and I have only the melancholly privilege of exhibiting to-day the very intimate union of the exterior surfaces of the gall-bladder and the duodenum, with only a trace of the cicatrix, left from the action of the ligature, apparent upon the mucous membrane of the duodenum, and without any indications of its effects in the cavity of the gall-bladder.

As you perceive, the agglutination is most thorough between the walls, so as to present the appearance of a solid, compact septum between the gall-bladder and duodenum, and thus affording the conclusive evidence of adhesive inflammation being developed by a single ligature, without having the surfaces joined by a circular suture of catgut.

Upon passing my probe through the opening from the sac into the cystic duct, you see it descends readily into this, and passes into one of the hepatic ducts, but cannot be made to enter the ductus choledochus, probably owing to the angle at which they unite.

I now introduce the probe from the opening of the common duct into the duodenum, and it is seen to glide readily into the tube, but does not find its way into the cystic duct.

There was evidently no obstruction to the bile, as, after laying open the duodenum, pressure upon the sac caused its escape at this orifice in its mucous lining, which is the outlet of the common duct.

It is an important feature in the history of this experiment that no undue inflammatory action was set up in the peritoneal cavity ; and, apart from the adhesion between the approximated walls of the sac and duodenum, with the attachment of the latter to the lower surface of the right lobe of the liver, and a slight adhesion of the stomach, as you perceive, to the under part of the left lobe, there remained no traces of peritonitis ; nor did the bile-ducts become involved in the inflammatory action, propagated from the ligature to the neighboring serous investments of the contiguous parts.

The second laparotomy, accomplished eight days after the first, while it might have been supposed that a predisposition to inflammation remained in the tissues, did not even result in adhesions with the line of suture through the long incision of the abdominal parietes, which was closed by the hair-lip suture needles passing through the edges of the peritoneal lining and the skin.

Your attention should be directed to a very material difference in the management of this case, from that of the previous experiments, by the sewing of a jacket around the body of the animal, which prevented any disturbance of the suture, that was torn out by most of the other dogs, thus leaving the cavity of the peritoneum exposed. Moreover, the animal of the last experiment was kept in an apartment adjoining my office, under my personal supervision, and supplied with food and water, regularly, from the beginning to the end of the traumatic phenomena, on each occasion. The inference I would have you make from these facts is, that the fatal results in the other experiments were owing to the lack of proper precautions for the treatment subsequent to the operation, and due likewise, in some of the cases, to the meddlesome surgery, by placing a circular row of stitches around the ligature that united the walls of the gall-bladder and duodenum, which is now proven to be entirely unnecessary, if not decidedly hurtful.

This demonstration would be complete, if it were alone intended to illustrate the mode of uniting these two parts ; but I will avail myself of the occasion to point out the various steps which may be proper in connection with my operation, and, though the order of things is reversed here, I have to state that, ordinarily, discharging the fluid contents by aspiration, or removing solid matters by incision, should precede this.

In all cases of supposed obstruction of the gall-duct, it would be proper to ascertain the practicability of effecting a passage through the natural channel before proceeding to make an artificial communication ; and if an effort to expel the fluid from the gall-bladder by compression proves ineffectual in cases of distension, it may be evacuated by a trocar having a tube so as to convey the bile or other fluid outside of the peritoneal cavity, when an incision through its walls will admit of the use of the finger for exploration.

I now proceed, without such preliminary step, to incise the wall of the sac with which the ligation of the duodenum was accomplished just now, and you see a dark liquid flowing out, which is more consistent than the natural bile, and hence we infer that it has remained beyond the ordinary period, so as to become inspissated. Upon passing my finger within its cavity no gall-stones or other solid matters are found ; and I now attempt to pass this silver probe into the cystic duct, but can discover no outlet where it should exist.

An examination of the duodenal extremity of the duct is next in order ; and by this incision through the wall of the canal, opposite the site of its entrance, I find the hardened termination of the duct covered over by the mucous membrane, which is now dissected off from it, but no opening in the duct can be discovered with the probe. It is evident, therefore, that the complete occlusion of both extremities of the tube leading from the gall-bladder to the duodenum is effected, and, upon passing my finger along the line of the common duct and the cystic duct, it gives me the conviction that the channel throughout is entirely obliterated, imparting a cord-like feel to the touch.

If our subject were alive, and presented similar pathological conditions, it is most likely that an artificial outlet for any collection in the gall bladder subsequently would best avail for the relief of the trouble; yet it would be proper to close the incision in the wall of the sac with the glovers suture of catgut, separate from the abdominal parietes, and then close the external wound with silk or silver wire suture, with the certainty that the ligature will effect an opening from the gall-bladder into the duodenum. I am so fully impressed with the prospect of a favorable result in a case suited to this operation, that it will be put into execution whenever an opportunity is afforded, and it may be remembered by those of you who are soon to graduate, and go out into your respective fields of practice, that should a patient come under your care with evident signs of obstruction of the gall-duct, it will be received gladly, either in my private surgical infirmary or in the Ivy Street Hospital located here, for operation by the ligation of the sac with the duodenum to open an artificial outlet for the bile.

With a view to have you act understandingly as to the diagnosis of such a case, let me state that all the tumours connected with the liver should be carefully considered. I have at the present time under my observation two enlargements of the left lobe of the liver, one being under my own direction, and the other under the care of a colleague in my private Surgical Infirmary, which occupy almost the entire epigastric region, extending nearly to the umbilicus. As these are more or less indurated, they could not be mistaken for the distended gall bladder. But in the event of an abscess forming in either, the diagnosis would be attended with great difficulty; and any purulent collection in the parenchymatous structure of the liver, when it extends below the margin of the ribs on the right side of the median line, is liable to be mistaken for a distended gall-bladder; or, on the other hand, the enormous proportions of the dilated sac may lead to the erroneous diagnosis of hepatic abscess. In the event that it extends down into the iliac region, as the dilated and elongated sac in dropsy of the gall-bladder has been known to do, it might be taken for an enlargement of the ovary, with fluid contents, and in all cases great caution is requisite in the diagnosis.

The element of fluctuation does not avail for a differential diagnosis, as it may exist in an abscess of the liver, in urinary infiltration from rupture of the ureter, or from an ovarian cyst, as well as in dropsy of the gall-bladder or other fluid collection in the sac. There may be, likewise, a semi-fluid accumulation in the dilated gall-bladder or a mass of a doughy consistence, from the inspissated bile, which imparts no sense of fluctuation, thus obscuring still more the indications connected with obstruction of the bile duct. But a simple and easily applied test for any fluid collection in the gall-bladder is the absence of any well-defined indurated margin on the lower outline of the tumor, and the continuation of the soft mass, with or without fluctuation, beneath the cartilages of the false ribs on the right side. If the fluid or pultaceous matter occupies the cavity of the sac, however much it may be dilated and extend downwards, it must necessarily reach up under the liver, which does not extend below the line of the costal cartilages unless hypertrophy of the organ exists.

The history of a case, in its gradual development and increase of dimensions, with the oblong outline, proceeding from beneath the ribs downwards, will very materially aid you in determining whether the gall-bladder forms the tumor or not. If, along with the local signs, you ascertain that the bile is absent from the fæcal evacuations, it will strengthen the presumption in favor of an accumulation in the gall-bladder; and this collection may be a fluid of disorganized biliary matter, of dirty dropsical form, of a purulent nature, of a clear muco-serous character, or of inspissated bile, with or without gall stones.

Having made out your diagnosis of obstruction in the duct, and a collection in the gall-bladder, a grave question is presented as to the propriety of any operative procedure. Should the occlusion of the gall-duct be accompanied with scirrhus of the pancreas, it would evidently preclude any proceeding short of the removal of this organ; and some of you will recall the patient in our hospital, who presented all the indications of such an affection, but was removed by his friends to die elsewhere, so that we were unable to make the autopsy for the verification of my diagnosis.

My conviction in regard to occlusion from impaction of gall-stones in the duct, or from agglutination of the sides of the canal without complete obliteration of the passages, is, that operative measures are likely to afford relief by removing the obstruction to the outlet of the bile. I am likewise impressed with the prospect of securing a salutary result when the ductus choledochus is permanently occluded and the bile still enters the gall-bladder and accumulates in the sac, by an operation such as has been described and demonstrated to you to-day in passing a ligature through the sac and duodenum. But if the case has run on until there is no more bile furnished by the liver, and only degenerated fluids are found in the sac, it seems to me that the occasion does not offer any encouragement for cholecystectomy, nor for cholecystotomy, as it has been undertaken, with a view to produce a fistulus discharge externally. You should see to it, then, that no case under your care be allowed to reach this hopeless condition; but seek to avert, by timely operation, the development of such results from obstruction of the gall-duct, and a favorable termination may be anticipated either by dilatation of the ducts or by establishing an artificial communication between the sac and duodenum.

What is the aim and end of this process has been shown by a series of experiments on dogs, published in detail by the Atlanta Medical and Surgical Journal, for September and October, 1884, and the results given in an elaborate article on Obstruction of the Gall-bladder and its Consequences, with Remedial Operation, for the October number of Gaillard's Journal.

---

IT is said that Dr. Koller, the discoverer of the anæsthetic properties of cocaine, has recently fought a duel. His antagonist, one of Billroth's assistants, received a wound that may prove fatal.—*Boston Med. and Surgical Journal.*

THE American Medical Association will meet in New Orleans on April 28th.

